# “Coronavirus EXPLAINED”: YouTube, COVID-19, and the Socio-Technical Mediation of Expertise

**DOI:** 10.1177/2056305120948158

**Published:** 2020-08-11

**Authors:** Nahema Marchal, Hubert Au

**Affiliations:** University of Oxford, UK

**Keywords:** YouTube, expertise, socio-technical mediation, content analysis, visual media

## Abstract

Since the coronavirus outbreak, YouTube has altered its content moderation policies to surface more “authoritative information” while removing videos that contain “medically unsubstantiated claims.” This was made urgent by incidents like a live-stream interview of renowned British conspiracy theorist David Icke—in which he falsely linked the spread of the coronavirus to 5G technology—that gained substantial traction online. Behind these events, however, lies a tension between the need for authoritative medical information and the socio-technical mediation that enables multiple, competing voices to lay claim to such authority on YouTube; a tension exacerbated by the current pandemic. Following an investigation into the sources and types of video content average users were likely to see when searching for information about the coronavirus on the site, we suggest that through its incentive structure and participatory affordances, YouTube may have subordinated expertise to a logic of likability—leaving institutional experts trailing behind.

“I never thought we’d have so many videos of hand-washing,” YouTube CEO Susan Wojcicki candidly remarked in an interview for CNN on 19 April. That day, Wojcicki announced the company would alter its content moderation policies to surface more “authoritative information” while removing videos that contain “medically unsubstantiated claims” (Humbrecht, 2020). Just 2 weeks prior, YouTube had already said it would tighten its grip around channels spreading misinformation after a live-stream interview of renowned British conspiracy theorist David Icke—in which he falsely linked the spread of the coronavirus to 5G technology—gained substantial traction online (Kelion, 2020).

Behind these events lies a deep tension between the societal need for authoritative medical information and the socio-technical mediation that enables multiple, competing voices to lay claim to such authority on a platform like YouTube; a tension exacerbated by the coronavirus pandemic. Indeed, while YouTube has arguably democratized knowledge production and skills sharing by providing any Internet user with a platform to share ideas;^1^ by virtue of its incentive structure and participatory affordances (Burgess & Green, 2009), it has also subordinated credible expertise to a logic of likability. And in the age of star influencers, social broadcasting, and search-engine optimized content, as we contend in this essay, this can leave institutional experts trailing behind.

With over 2 billion monthly active users, YouTube is a social media behemoth and a key player in the contemporary information ecosystem. It is one of the largest search engines in the world, second only to Google by volume, and a gateway to the news for a large number of its users (Burgess & Green, 2009, p. 16). In recent years, it has also emerged as a major source of information about science, technology, and health, especially for young people (Anderson & Jiang, 2018). According to a 2019 survey, around three-quarters of US adults and 90% of 18- to 24-year-olds say they use the site (Van Kessel, 2019). In the United Kingdom alone, an estimated 35.6 million people visit YouTube each month, and the average adult spends about half an hour on it every day, making it the most popular online video platform in the country (Ofcom, 2019), and viewership has been skyrocketing during the pandemic.

Given the centrality of YouTube as an information hub, in March 2020, we decided to investigate the type and quality of content average users were likely to come across when searching for information about the coronavirus on the site (Marchal et al., 2020). We were particularly interested in finding out what sources and channels were most represented in search results; to what extent video content around the coronavirus’ origin, transmission, and cure was being politicized; and how much of it was factually inaccurate, misleading, or conspiratorial. Finally, we wanted to get a sense of how much attention and engagement different types of videos were receiving.

Like many other social media platforms, YouTube is powered by a search algorithm and recommender system based on the principle of “collaborative filtering” (Covington et al., 2016), designed to help users navigate the millions of pieces of content available on its site. Although little is known about the precise workings of its proprietary algorithm, this has been suggested to mean that content surfaces based, in part, on other users with similar tastes and preferences. Research also indicates that YouTube users rarely scroll past the top 20 results when searching for content, and company executives have suggested that recommendations are responsible for 70% of time spent on the site (Rodriguez, 2018). In an effort to replicate this basic user experience, we thus honed in on the first 20 video results returned for a search query, as well as what YouTube calls the most frequent “related” videos displayed in the sidebar.

Using Google Trends, in early March, we first collected a list of the 10 most popular search queries entered into the YouTube search bar in the United Kingdom related to “coronavirus” since January 2020. We settled on four popular coronavirus-related search terms in the United Kingdom—“coronavirus UK,” “coronavirus China,” “coronavirus symptoms,” and “coronavirus conspiracy”—as these covered a wide range of topics reflecting strong public interest.^2^ We then first queried the YouTube API’s search function with our four search terms on 20 March 2020 and selected the top 20 video results provided by YouTube in the order of “relevance”— the default metric for this parameter—as well as the top 60 related videos for each search term.^3^ Our final sample thus consisted of 320 videos, 80 per search term.

All videos and associated channels in our sample were reviewed in depth and manually classified by the authors, who achieved high inter-rater reliability following two rounds of training and test coding (Krippendorf’s α = .803). A heuristic approach was taken to content classification based on the type of evidence marshaled, degree of politicization, and factual accuracy of the information presented. Videos were classified as factual and neutral when they featured high-quality reporting on the coronavirus pandemic, which included factoids and news reports from professional news organizations and public health agencies. Videos relaying verifiably false information or conspiracy theories about the origin, transmission, and treatment of the coronavirus; trutherism; xenophobia and denial of mainstream scientific positions as assessed against World Health Organization (WHO) public advisory information were coded as junk and conspiratorial. Personal and testimonial videos discussed the coronavirus pandemic from a personal or testimonial point of view, such as patients describing their symptoms, first-person accounts or recommendations, debunking efforts from independent vloggers and health practitioners, as well as undercover investigations and talk shows. Politicized videos, finally, were ones in which the coronavirus pandemic, its provenance, spread, as well as the efficacy of various government responses were discussed or debated from a political perspective. This included political comedy shows, debates, podcasts of a political nature, and ideologically motivated fact-checks.

Channels themselves were classified as either falling into one of the five following categories: government and public agencies, for the official channels of government agencies and international bodies, such as the US White House or United Nations; independent content creator, for channels of independent vloggers, media commentators, health professionals, and educators; professional health, for domestic and intergovernmental public health agencies, hospitals, and professional health websites, such as the WHO, the National Health Service (NHS), or WebMD; professional news, for established news organizations, broadcasters, digital and print media outlets; and finally, state-backed media, for channels of media organizations that are either directly funded by the state and are editorially controlled by their respective governments.

Results from this content analysis were, to a certain degree, reassuring. Only a small fraction of the videos we analyzed were found to be peddling unsubstantiated claims^4^ on the virus’ origins, transmission patterns, or cure (less than 2%). The ones that did tended to relay sinophobic tropes and amplified snippets of misinformation that had already been debunked elsewhere (Andersen et al., 2020), such as claims that the virus had been unleashed from a Wuhan lab in a geopolitical ploy orchestrated by the Chinese Communist Party. In contrast, factual and balanced reporting mostly dominated video results, especially for searches linked to “coronavirus UK,” where fully 80% of top 20 results returned professional news report.

However, this only tells part of the story. Our analysis shows that while instances of junk and highly politicized health news and information were minimal, this content received far more engagement in the form of comments than any other type of videos: around 9,000 comments per million views. Another arresting finding was the channels of public health institutions such as the WHO and NHS were rarely, if ever, returned in our search results. Instead, four-fifths of the channels sharing coronavirus news and information in our dataset were maintained by news outlets and independent content creators, including health practitioners and commentators who took it upon themselves to translate the latest scientific evidence and governmental policies to their audiences.

This once again underscores the core tension between the attention economy and cautious, deliberate expertise. For a long time, public health and science communication was the realm of scientists and professional reporters, who made sporadic apparitions to communicate around key issues. The advent of the Internet, however, has broken existing institutional barriers and enabled new forms of mediated authenticity; a process to which YouTube has been central. The video-sharing platform has made it easier to present oneself as an expert and gain a reputation for credibility and trustworthiness among a dedicated fan base (Lewis, 2020; Marwick, 2015). One can achieve this without relying on the apparatus typically required of professional print or digital media news outlets, thus reshaping who can be regarded as an “authoritative” voice.

Through its participatory culture, YouTube also invites “synthetic personalization” (Fairclough, 2010): the creation of a sense of intimacy and relatability between creator and audience through a fictitious dialogue. Videos returned for “coronavirus symptoms” in our sample, for instance, tended to revolve around personal stories—such as firsthand testimonials from COVID-19 survivors or recommendations by private citizens and doctors around social distancing. Among the highly politicized videos identified through our content analysis—many of which were returned through searching for “coronavirus China” or “coronavirus conspiracy”—the most frequently recommended ones consisted in podcasts and comedy shows, followed by “exposés” in which vloggers purported to reveal cover ups about the pandemic that experts or political leaders had kept privy. In their description and titles, these YouTube commentators often used excessive capitalization and discursively distanced themselves from an elusive “other”—whether the mainstream media, the political elites, or doctors—which they portrayed as enemies of truth.

As the coronavirus pandemic continues to sweep across the globe, ensuring access to reliable and trustworthy public health information has proved as challenging as curtailing the spread of the virus itself. Speaking at the Munich Security Conference on 15 February 2020, the WHO director Dr. Tedros Adhanom Ghebreyesus warned against the consequences of an overabundance of more or less accurate information that would make it difficult for citizens to find reliable guidance when needed (World Health Organization, 2020). The real challenge during this crisis, his address underscored, was not to keep people informed so much as to help them navigate the sheer volume of novel information and claims made available every day. These warnings have proved all the more prescient now that health misinformation is having real, nefarious consequences on the public: one only has to look to recent reports of individuals ingesting chloroquine phosphate—a toxic chemical erroneously touted as a potential remedy for COVID-19 (Piller, 2020).

At a time when individuals and institutions compete to create new circuits of knowledge, any actor seeking influence or profit can readily exploit information gaps or strategically mobilize claims from reputable sources (Golebiewski & boyd, 2019) and take them out of context to serve the agenda that an audience expects of them. As the Director of Infectious Hazards Management at the WHO’s Health Emergencies Programme, Sylvie Briand, said, “it is a new challenge [. . .] you need to be faster if you want to fill the void” (as cited in Zarocostas, 2020). Under this “consensus of the most liked” (DiResta, 2020), the most authoritative information—in our case, that of public health officials—is neither the most appealing to social media users nor the most rewarded by the dictates of algorithmic curation. Therein lies the core paradox of social media mediated authority; one that no amount of policy changes can bandage over.

## Notes

1. Although, in this respect, it is critical to consider the politics of algorithmic visibility on social media in general and on YouTube in particular (Bucher, 2012; Horak, 2014; Rieder et al., 2018).

2. For comparison, we also compiled a list of auto-complete suggestions for the term “coronavirus” in the YouTube GB search bar, using a Google Chrome browser in Incognito Mode. The term “coronavirus” was selected over “COVID-19” as it was by far the most popular search term at the time of data collection.

3. There are, of course, multiple caveats to this approach. The videos captured in our sample only provide a snapshot of what was being surfaced on YouTube at a specific point in time and do not account for the personalization or localization of search rankings. Likewise, due to the black-boxed nature of YouTube’s algorithm, we are also necessarily limited in the kind of claims we are able to make about the representativeness of the sample collected. Such limitations are inherent to studying socio-technical systems where information visibility is determined by technical, temporal, and social dynamics in constant flux (Arthurs et al., 2018).

4. The science on COVID-19 is itself evolving and claims were evaluated against the scientific consensus at the time of our data collection.
